# Decision making influences movement variability and performance of high-level female football players in an elastic resistance task

**DOI:** 10.3389/fpsyg.2023.1175248

**Published:** 2023-09-15

**Authors:** Sílvia Tuyà Viñas, Bruno Fernández-Valdés Villa, Carla Pérez-Chirinos Buxadé, Jacob González, Gerard Moras Feliu

**Affiliations:** ^1^Department of Sports Performance, Institut Nacional d’Educació Física de Catalunya (INEFC), Universitat de Barcelona (UB), Barcelona, Spain; ^2^Department of Health Sciences, Research Group in Technology Applied to High Performance and Health, TecnoCampus, Universitat Pompeu Fabra, Barcelona, Spain; ^3^Department of Strength and Conditioning, Futbol Club Barcelona, Sant Joan Despí, Spain

**Keywords:** decision making, resistance training, movement variability, inertial measurement unit, entropy, performance, passing accuracy, football

## Abstract

**Introduction:**

The inclusion of sport-specific constraints in resistance training promotes the development of player abilities in an integrated way, which maximises the effectiveness of player adaptations induced by training. Considering that perceptual-cognitive abilities play a fundamental role in football, decision making could be introduced to enhance the cognitive similarity of resistance tasks to sport actions. However, it is unknown how decision making as a constraint could affect the player during an elastic resistance task. Therefore, the aim of this study was to investigate the effects of decision making of high-level female football players on movement variability and performance during an elastic band resistance task.

**Methods:**

Twenty-three high-level female football players performed the elastic resistance task with a ball, both as attackers and as defenders without decision making (NDM) and with decision making (DM). The movement variability was quantified using the sample entropy derived from the acceleration recorded with an accelerometer placed at the lower back of each player. The passing accuracy of the attacker was quantified using a scoring scale.

**Results:**

Results revealed that adding decision making to an elastic resistance task increased the movement variability of the defender but did not affect the movement variability of the attacker. In contrast, the passing accuracy of the attacker was reduced. Overall, the attacker had a higher movement variability compared to the defender.

**Discussion:**

These findings suggest that decision making, as a football-specific constraint, can enhance the potential of an elastic resistance task in training. This is due to the fact that it reduces control and regularity of movement for the defensive role player and increases technical difficulty for the attacking role player. Furthermore, these effects are beneficial, as they can promote the adaptive processes necessary to optimise the performance of the players.

## 1. Introduction

In recent years, football has become a very physical sport, as players perform a large number of high intensity actions that require high muscular power (di Salvo et al., 2009). Moreover, for these actions to be executed effectively, players should process multiple inputs from a dynamic and changing environment (Araújo et al., 2006). To enhance player performance in this context, it is necessary to integrate the diverse abilities inherent to the sport into resistance training, thereby optimizing the transfer of strength gains to sport actions (Suarez et al., 2019). Among the pivotal considerations, ensuring the dynamic correspondence of exercises stands as a fundamental factor (Araújo et al., 2007; Suarez et al., 2019). Dynamic correspondence, as outlined by Verkhoshansky and Siff (2009), refers to the ability of an exercise or training program to directly affect the athletes’ sport performance, due to its similarity or specificity to sport skills—commonly known as the “transfer effect”. In addition, the training principles of overload and variation must also be ensured. Collectively, these three principles constitute the foundation of the structure of levels of approximation proposed by Moras (1994), which defines a method for organizing exercises into levels from lower to higher dynamic correspondence (Fernández-Valdés Villa, 2020). Moreover, constraints can be categorized into task, individual, and environmental classes. In sport, task constraints refer to those elements that alter or restrict its execution, either through rules or added equipment (Newell, 1986). In addition, several strength and conditioning coaches use this constraint-led approach, integrating constraints specific to the sport context for this purpose, which is also highly supported by the scientific literature (Newell, 1986; Araújo et al., 2007; Davids et al., 2008). In this regard, the interaction with the ball (Moras et al., 2018; Makhlouf et al., 2022) or decision making (Gil-Arias et al., 2016; Natsuhara et al., 2020) are some football-specific constraints, while external resistance as elastic bands, dumbbells or a weighted vest are some nonspecific constraints.

Resistance training using elastic bands allows exercises with different amplitude and direction of movement (Verkhoshansky and Siff, 2009), and with different velocities (Janusevicius et al., 2017). This makes it possible to respect the dynamic correspondence of the exercises with sports movements, and to incorporate football-specific constraints into them. As a result, there has been a growing trend in the use of elastic band exercises, especially performed on the playing field itself. However, their use in resistance training does not usually follow the dynamics of effort, rate and time of maximum force production, and regime of muscular work (Verkhoshansky and Siff, 2009). In any case, this type of training has been shown to achieve similar strength gains to those achieved with conventional equipment, such as free weights or weight machines. Overall, studies on elastic bands have analyzed the effects of this equipment through different tests, assessing strength, power or muscle activity, and by using linear analyses such as mean, standard deviation (SD) or coefficient of variation (Bergquist et al., 2018; Lopes et al., 2019). To the best of our knowledge, we are not aware of any studies that have analyzed the elastic resistance training using non-linear systems, which can provide information about the structure of a signal.

In recent years, the ball has been incorporated into some resistance exercises as a specific constraint, with some studies analyzing its effect through non-linear systems. The findings revealed that the interaction with the ball involves greater anticipatory and compensatory adjustments of the body, due to the external perturbation of posture that occurs when players orientate their trunk to catch the ball, throw the ball, or make a pass (Moras et al., 2018). Besides, perceptual-cognitive abilities, which affect decision making processes, also play a major role in sport, as they are directly related to individual and team success (Bar-Eli et al., 2011). One of the basic scenarios in football in which decision making takes place is 1vs.1. In this situation, the attacker usually attempts to dribble or deceive the defender in order to pass the ball or advance on the field, avoiding the opponent stealing the ball. To achieve this, the player needs to disrupt the stability of the dyadic system they form together (Davids et al., 2006). Decision making in team sports is based on a continuous process of exploration and selection of relevant information (Bar-Eli et al., 2011). To ensure natural behavior, the task should have an experimental design as representative as possible of the conditions of the environment it is intended to simulate (Araújo et al., 2007). In general, most of the investigations examining decision making used video clips of real game situations, so that when the video was stopped, the participant could decide among the game options (Natsuhara et al., 2020; de Waelle et al., 2021); other research analyzed parameters related to decision making based on videos of match situations, such as passing angles, shooting angle and interpersonal distance (Corrêa et al., 2016); and others used videos of training match situations for participants to analyze their own decision making in an attacking action using open-ended questions (questioning) about the context of the action, the possible solutions, the selected response, and the results of the decision (Gil-Arias et al., 2016). So far, no research has been found that includes decision making in exercises with elastic resistance, which may provide a higher cognitive similarity to the task and greater representation of the sport context.

The way in which constraints cause postural destabilization to players can be measured through movement variability (MV). In this case, MV refers to execution variability, which describes the adjustments of the body between repetitions of an exercise (Cowin et al., 2022). Thus, it assesses movement regularity, and it is an indicator of motor control (Davids et al., 2003). When the player faces tasks with constraints, this can either lead to a greater effort to perform the task and, consequently, increase the difficulty, or it can increase the cognitive load, thus increasing the complexity of the task. The central nervous system, through perceptual-motor operations, explores different options offered by the degrees of freedom to find an optimized motor solution, which causes an increase in its MV (Schöllhorn, 2000). Building on this perspective, MV could describe the process of eliciting stable motor responses, providing valuable insights into the player’s coordination features, as well as shedding light on the dynamics of the task (Newell et al., 1989; Mehdizadeh et al., 2015; Dhawale et al., 2017). Moras et al. (2018), in their study, demonstrated that the incorporation of a rugby-specific ball pass to a movement with inertial resistance produced an increase in the MV of the athletes. Moreover, in the situation in which the body is not able to manage the movement optimally, there is a decrease in performance (Stergiou et al., 2006). However, the relationship of MV and performance is unclear (Urbán et al., 2019) and it might depend on the nature of the intrinsic dynamics of the system and the task constraints, as suggested by Newell and Vaillancourt (2001). Nevertheless, it seems that after a period of training, an adaptation process occurs, leading to an improvement in performance and a reduction in MV (Fernández-Valdés et al., 2020; Bashford et al., 2022), indicating that motor learning takes place (Newell et al., 1989).

The analysis of MV has been approached from different perspectives (Cowin et al., 2022), one of which involves linear analysis systems using the SD or the coefficient of variation of time series data (Bashford et al., 2022). Another approach is the non-linear analyses based on entropy, which are suitable for dealing with the complexity of biological systems, as it quantifies the amount of regularity and unpredictability of point-to-point fluctuations across different time scales (Harbourne and Stergiou, 2009). Both types of analysis can provide information on the magnitude of variability and the structure of variability, although variation in how a motor behavior emerges in time is best captured by tools developed for the study of non-linear systems (Stergiou and Decker, 2011). Among the measures of entropy, sample entropy (SampEn) is one of the most widely used in sport and health sciences (Richman and Moorman, 2000; Murray et al., 2017; Moras et al., 2018; Fernández-Valdés et al., 2020, 2022). It is a method to describe the regularity and predictability of a time series, comparing patterns within a specified window (Richman and Moorman, 2000). Approximate entropy is a similar measure of complexity as SampEn, comparing patterns within a fixed window, but is less influenced by noise. Nevertheless, it is highly sensitive to the length of the time series data, as the estimation of pattern probabilities becomes less accurate with fewer data points. Instead, multiscale entropy calculates entropy at multiple scales, which allows understanding the dynamics of the system and detecting changes. However, it has a higher computational complexity, which can limit its practicality when dealing with large datasets. It divides the signal into coarse-grained time series and calculates the entropy at each scale (Costa et al., 2002). In general, SampEn is more robust to variations in time series length and can provide meaningful insights even with smaller datasets (Pincus, 1991). Entropy has been used to describe pathological conditions (Costa et al., 2002), changes in postural control (Lubetzky et al., 2015; Williams et al., 2016), assessment of running (Murray et al., 2017), tactical behavior in football (Santos et al., 2018), or movement regularity in resistance training tasks (Moras et al., 2018; Fernández-Valdés et al., 2020). In recent years, one of the methods to measure the MV has been approached by the calculation of entropy from the acceleration data collected at the lower back, using an inertial measurement unit (IMU) (Murray et al., 2017; Moras et al., 2018; Fernández-Valdés et al., 2020). This option makes it possible to quantify the MV of the athlete with data collected directly from the body, using a variable obtained directly from the accelerometer without the need for mathematical calculations. Furthermore, this location of the IMU can describe the global movement of the body due to its proximity to the center of mass (Montgomery et al., 2010).

In this regard, no research has been found that has evaluated the effect of decision making on the MV and performance of the athlete in a task with elastic resistance. This aspect would provide relevant information to understand the behavior of the body at a coordinative level and the influence on task performance when constraints involving perceptual-cognitive capacities are present in a resistance task. Therefore, the aim of this study was to investigate the effects of decision making of high-level female football players on MV and performance during an elastic band resistance task. Considering the fact that the incorporation of constraints in the task, such as decision-making and/or interaction with the ball, could lead to an increase in MV and, consequently, a decrease in performance, we hypothesized that (a) the attacking and defensive roles will have higher MV in the decision making condition (DM) compared to the no decision making condition (NDM), (b) in NDM and DM, the attacker will have higher MV compared to the defender, and (c) the attacker will reduce her passing accuracy in DM compared to NDM.

## 2. Materials and methods

### 2.1. Participants

The study involved 23 high-level female football players, all from the same club ((mean ± SD) age: 22.65 ± 5.16 years, height: 1.67 ± 0.64 m, body mass: 59.75 ± 14.08 kg). Twelve of the players belonged to the first team of the club, competing in the first women’s division in Spain, Liga Iberdrola, and in the UEFA Women’s Champions League in the season in which the data were taken (2021–2022); the remaining 11 players belonged to the second team of the club, competing in the second women’s division in Spain, currently called Primera Federación Femenina. All players were informed of the benefits and risks of the study before signing the informed consent. All procedures complied with the Declaration of Helsinki (2013) and were approved by the Ethics Committee of the Catalan Sports Council (036/CEICGC/2021).

### 2.2. Data Collection

Data collection was conducted in 10 sessions during the competitive season. All the sessions were performed on the highest load day of the regular training week (three days before the match day) during the strength training session and before the football technical training session. The research task was included as part of a random order strength circuit and it took place on the field, on natural grass. Therefore, the players wore their usual football boots. In the first session, a pilot test was conducted with two players from the first team to check the task design.

In the data collection sessions, the players first performed a warm-up protocol led by the strength and conditioning coach of the team, which was identical in all sessions. Afterwards, an IMU device with an accelerometer (WIMU, Realtrack Systems, Almeria, Spain: weight: 0.07 Kg, size: 0.081 m × 0.045 m × 0.016 m) was attached to the lower back of the players, at the L4-L5 level, using an adjustable rigid belt. The IMU was set to a sampling frequency of 1,000 Hz and it was calibrated on a flat surface. A 4 K camera (Sony FDR-AX53) recording at 60 Hz was used to synchronize the accelerometer signal of the IMU with the video of each session for a further visual checking.

**Figure 1:**
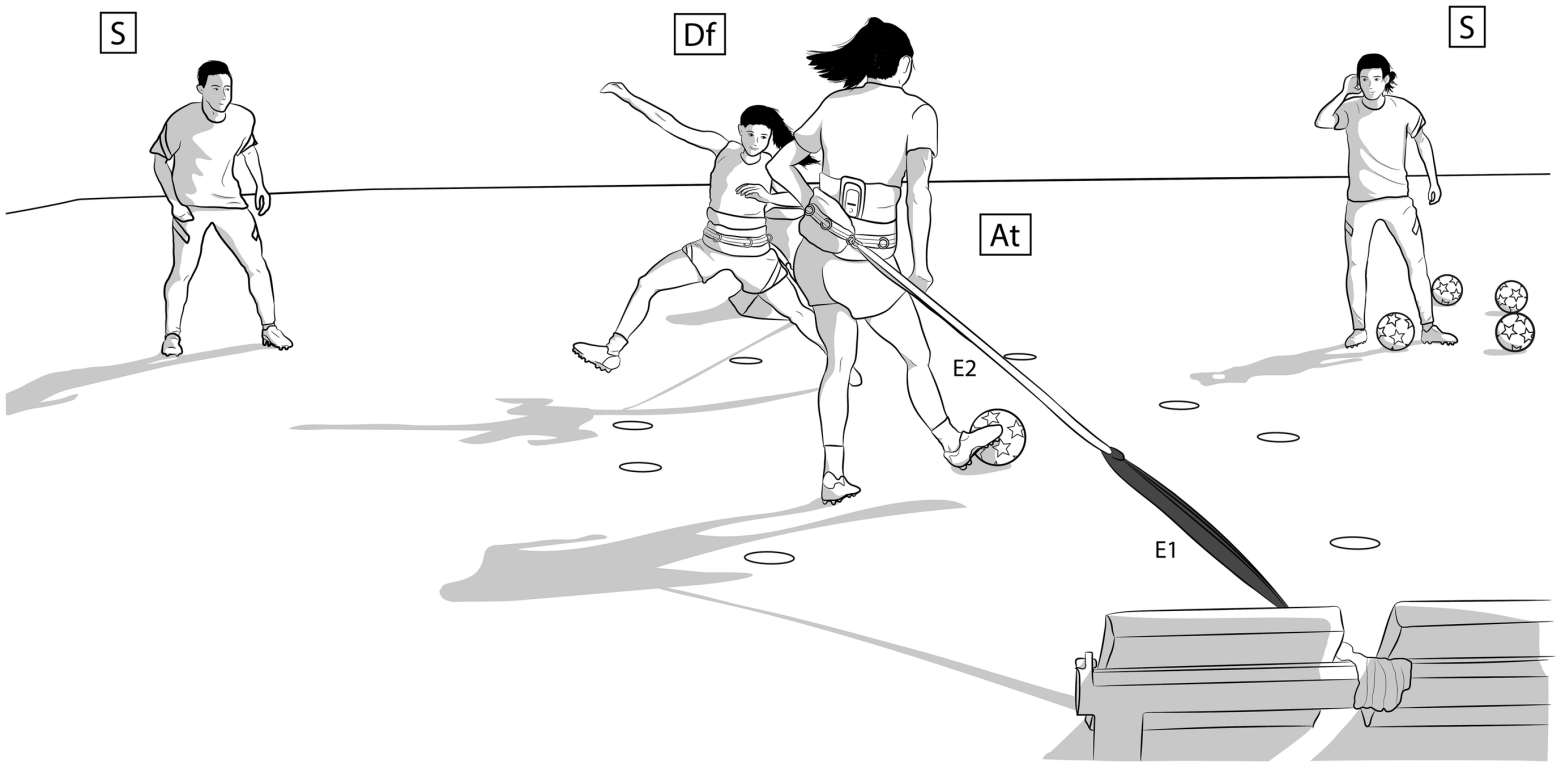
Design of the task. At, attacker; Df, defender; S, staff member; E1, elastic band 1, of higher resistance; E2, elastic band 2, of lower resistance.

The research task was classified as a level 2 task from the structure of levels of approximation, which corresponds to a medium level of specificity (Moras, 1994), as a low external resistance was applied to a task with certain dynamic correspondence to some football actions. The task was performed in pairs, one player with the role of attacker and the other with the role of defender, both players facing each other. Two staff members positioned in front of the attacker, one on each side of the defender, were in charge of passing and receiving the ball. Both attacker and defender moved forward and backward at the same time within the delimited space, with an elastic resistance attached behind their waist. The attacker received the ball when she reached the most advanced point of the movement and she had to pass the ball to one of the staff members with a single touch. The task was repeated multiple times. In each repetition, the attacker received the ball alternately from each side. Two conditions of the task were conducted, without decision making (NDM) and with decision making (DM). In the NDM condition, the attacker received a voice command from one of the researchers when she was at the rearmost point of the movement indicating the side to return the ball. In this case, the defender did not participate actively, but only had to move forwards and backwards in synchronization with the attacker. On the other hand, in the DM condition, the defender had the objective of intercepting the ball after the attacker had passed it, making a real active defense. The attacker had the objective to get the ball to one of the two staff members, so she had to decide the best passing option. In this case, the DM condition of the task simulated a 1vs.1 situation of the game. In both conditions, NDM and DM, the attacker was asked to move as fast as possible, and no instructions were given about which foot she should contact the ball with (see Figure 1).

Referring to the elastic resistance, two circular elastic bands joined together were used for each player. The elastic band of higher resistance was attached to a fixed point close to the ground, and the elastic band of lower resistance was attached to the waist of the player, using a training belt and a carabiner. The movement space of the players was delimited with flat cones, so that the back boundary coincided with the beginning of the deformation of the elastic ensemble and the front boundary with a deformation equivalent to 150 N, resulting in a space of 2 m, measured *in vitro*. A maximum force of 150 N was selected, as it corresponds to a low external resistance that made it possible to perform a movement with a specific technical component (i.e., ball passing). The separation between the players when they were both at their forward boundary was 0.5 m. The two staff members were positioned at the level of the point at which the elastic band of the defender was attached to the support structure, with 8 m distance between them.

Each player performed a total of 16 sets of 12 repetitions of the task, 8 sets as an attacker and 8 as a defender, always with a different partner. For each role, 4 of the sets were made in the NDM condition and 4 in the DM condition, in random order. In each data collection session, each player only performed 2 sets of each role. In NDM, the passing direction of the attacker was also randomly pre-set, although the same number of passes to the right as to the left (6 and 6) was ensured.

Eleven of the 12 repetitions of each set were selected, always discarding the last one, where the intensity of the movement normally decreased. On the one hand, for the analysis of MV, the acceleration signal from the data obtained with the accelerometer of the IMU was used, which corresponded to the module of acceleration in the three coordinate axes. The SPRO 951 software (version 1.0.0, Realtrack Systems, Spain) was used to process the acceleration signal and synchronize it with the video. The acceleration data were not filtered to accurately analyze the variability within the time series, as Craig et al. (2020) did, following the recommendation of Mees and Judd (1993). The SampEn of the acceleration signal of each set was calculated based on the algorithm presented by Richman and Moorman (2000). A preliminary analysis confirmed that the results of SampEn calculated on the raw acceleration signal were consistent with those calculated on downsampled data, such as to 200 Hz. On the other hand, to calculate the task performance of the attacker, the accuracy of her passes was analyzed using the mean repetition score. Each pass was scored with 0, 1 or 2 points, depending on the minimum distance between the ball, i.e., ball trajectory, and the point where the receiving staff member was at the instance when the ball was released from the foot of the attacker. For this purpose, a video analysis was performed using Kinovea software (version 0.9.5). The criteria for scoring were 2 points for a distance (d) of d ≤ 1 m, 1 point for 1 < d ≤ 2 m and 0 points for d > 2 m. The mean repetition time and the root mean square of the acceleration without gravity (RMS ACC) were calculated to check whether there were differences in the duration and magnitude of acceleration between the sets.

### 2.3. Statistical analyses

Continuous variables were summarized as mean and standard deviation (see Supplementary material). Since the data were not normally distributed (Shapiro–Wilk test was performed), they were log-transformed. Subsequently, to analyze the effects of the player role (attacker/defender), the decision making condition (DM/NDM) and the interaction of both factors in each response variable (MV, RMS ACC and mean repetition time), a linear mixed model was performed, where participant was the random effect. The degrees of freedom were corrected using the Kenward-Roger method (Kenward and Roger, 1997). Additionally, comparisons were performed within factors and were assessed via effect size (ES) using 95% confidence intervals. Thresholds for ES were: 0.2 trivial; 0.6 small; 1.2 moderate; 2.0 large; and > 2.0 very large. Finally, regarding the passing accuracy of the attacking player, the comparison between decision making conditions was assessed by a Wilcoxon-signed rank test, and Pearson correlation was performed to analyze the relationship between MV and passing accuracy. Differences between the first and second team players in MV and passing accuracy were tested using Student’s t-test for independent samples. For all statistical tests, a nominal significance level of 5% (value of *p* < 0.05) was applied. The statistical analysis was performed using R-Gui (v4.1.2, The R Foundation for Statistical Computing, Vienna, Austria).

## 3. Results

The MV obtained by the female football players was described with the SampEn (mean ± SD). The MV of attackers was 0.170 ± 0.017 arbitrary units (a.u.) in NDM and 0.174 ± 0.013 a.u. in DM, and the MV of defenders was 0.134 ± 0.010 a.u. in NDM and 0.157 ± 0.019 a.u. in DM. The passing accuracy of attackers was 1.674 ± 0.178 points in NDM and 1.553 ± 0.177 points in DM. Regarding the RMS ACC, attackers obtained 0.155 ± 0.064 g-force (g) in NDM and 0.148 ± 0.048 g in DM, whereas defenders obtained 0.143 ± 0.055 g in NDM and 0.135 ± 0.060 g in DM. The mean repetition time of the attacker was 1.884 ± 0.116 s (s) in NDM and 1.895 ± 0.140 s in DM, and that of the defender was 1.877 ± 0.118 s in NDM and 1.872 ± 0.149 s in DM. The MV values obtained for each team were for the attackers of the 1st team 0.167 ± 0.017 a.u. and for those of the 2nd team 0.178 ± 0.014 a.u.; and for the defenders of the 1st team 0.142 ± 0.018 a.u. and for those of the 2nd team 0.151 ± 0.019 a.u. Regarding the passing accuracy of the attackers, the first team obtained 1.592 ± 0.142 points and the second team 1.612 ± 0.113 points.

**Figure 2:**
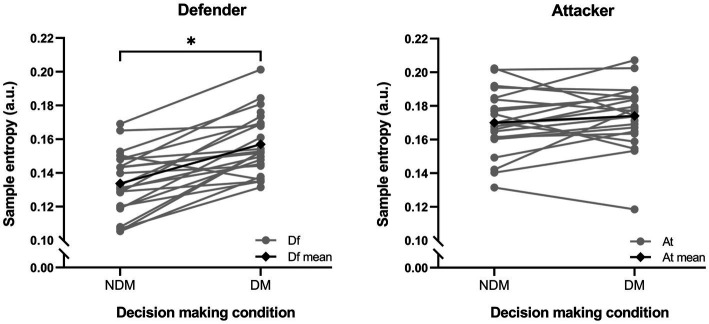
Individual movement variability described using sample entropy of the attacker and defender roles and the mean of the roles, comparing the no decision making (NDM), and decision making (DM) conditions. The significant differences are shown as * (*p* < 0.001). At, attacker; Df, defender; a.u., arbitrary units.

**Figure 3:**
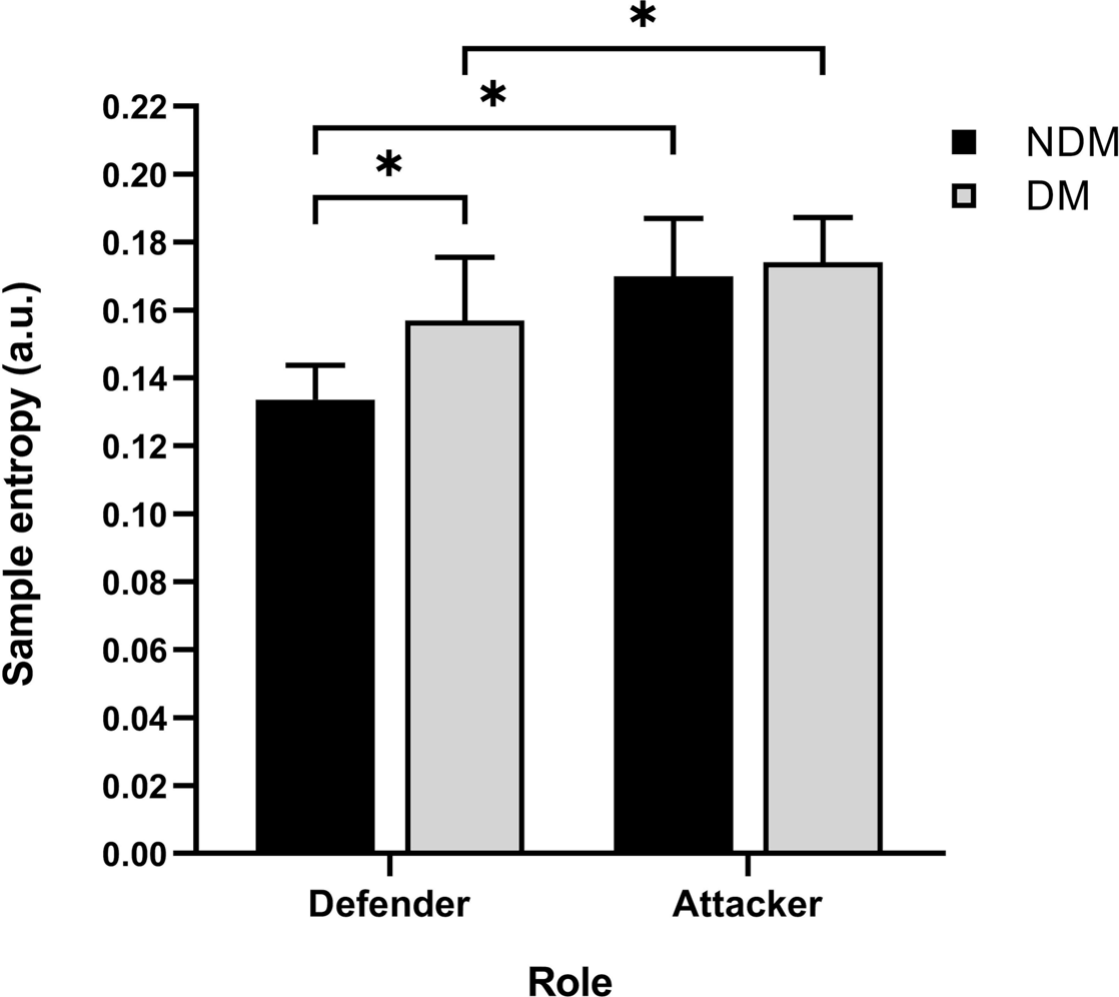
Comparison of movement variability described using sample entropy between decision making conditions and roles. It shows the sample entropy values (mean and 95%CI) of the defender and the attacker low back acceleration signal in the two decision making conditions: no decision making (NDM) and decision making (DM). Significance values come from the repeated measures model with log-transformation and only statistically significant differences are shown as * (*p* < 0.001). a.u., arbitrary units.

Considering that the data were not normally distributed, modeling was done with the logarithmic transformation of the MV, RMS ACC, and mean repetition time variables. The two main factors of the model using the log(MV) variable (decision making condition and role) were found to be statistically significant (*F* (64.230) = 25.968, *p* < 0.001; *F* (64.248), *p* < 0.001, respectively). In addition, the interaction using the log(MV) between both factors was significant (*F* (64.262) = 14.204, p < 0.001). Differences between NDM and DM were found in the defensive role (p < 0.001) but not in the attacking role (*p* = 0.352) (see Figure 2). Moreover, there were differences between the defender and the attacker in both conditions of decision making (*p* < 0.001 in NDM and in DM) (see Figure 3). There was no interaction between the two factors using the log(RMS ACC) and log (mean repetition time) variables (*p* = 0.814 and *p* = 0.259, respectively). These results are explained below in accordance with the research hypotheses.

**Figure 4:**
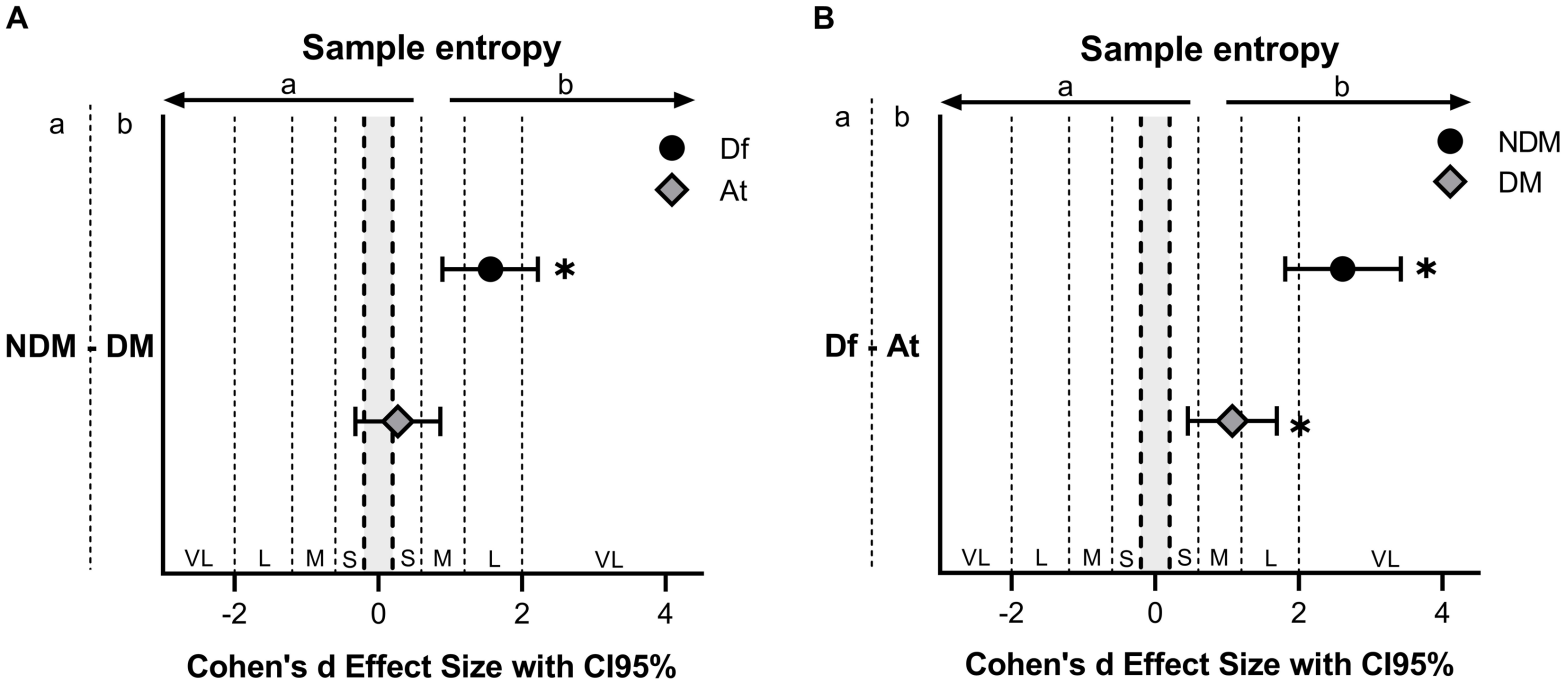
Effect Size using Cohen’s *d* of the sample entropy differences between decision making conditions **(A)** and player roles **(B)**. The error bars indicate the uncertainty in the changes of the average with a 95%CI. * indicates significant differences (*p* < 0.001). NDM, No decision making; DM, decision making; Df, defender; At, attacker; S, small; M, moderate; L, large; VL, very large; CI, confidence interval.

As mentioned above, due to the significance of the interaction between decision making condition and role, when players took the attacking role, no differences in MV were found between NDM and DM, with a small ES (p = 0.352, ES = 0.27, 95%CI: −0.32 – 0.86). On the other hand, when players took the defensive role, the MV was higher in the DM condition than in NDM, with a large ES (*p* < 0.001, ES = 1.56, 95%CI: 0.89–2.22) (see Figure 4). Regarding the RMS ACC and the mean repetition time, there were no differences between NDM and DM in any of the player roles, as the interaction between decision making condition and role was not significant for these variables.

Due to the significance of the interaction between decision making condition and role, in both decision making conditions the attacker showed higher MV compared to the defender, with a very large ES in NDM (*p* < 0.001, ES = 2.61, 95%CI: 1.81–3.42) and moderate in DM (*p* < 0.001, ES = 1.07, 95%CI: 0.45–1.69) (see Figure 4). Despite this, the RMS ACC and the mean repetition time showed no differences between attacker and defender in any of the decision making conditions, as the interaction between decision making condition and role was not significant for these variables.

**Figure 5:**
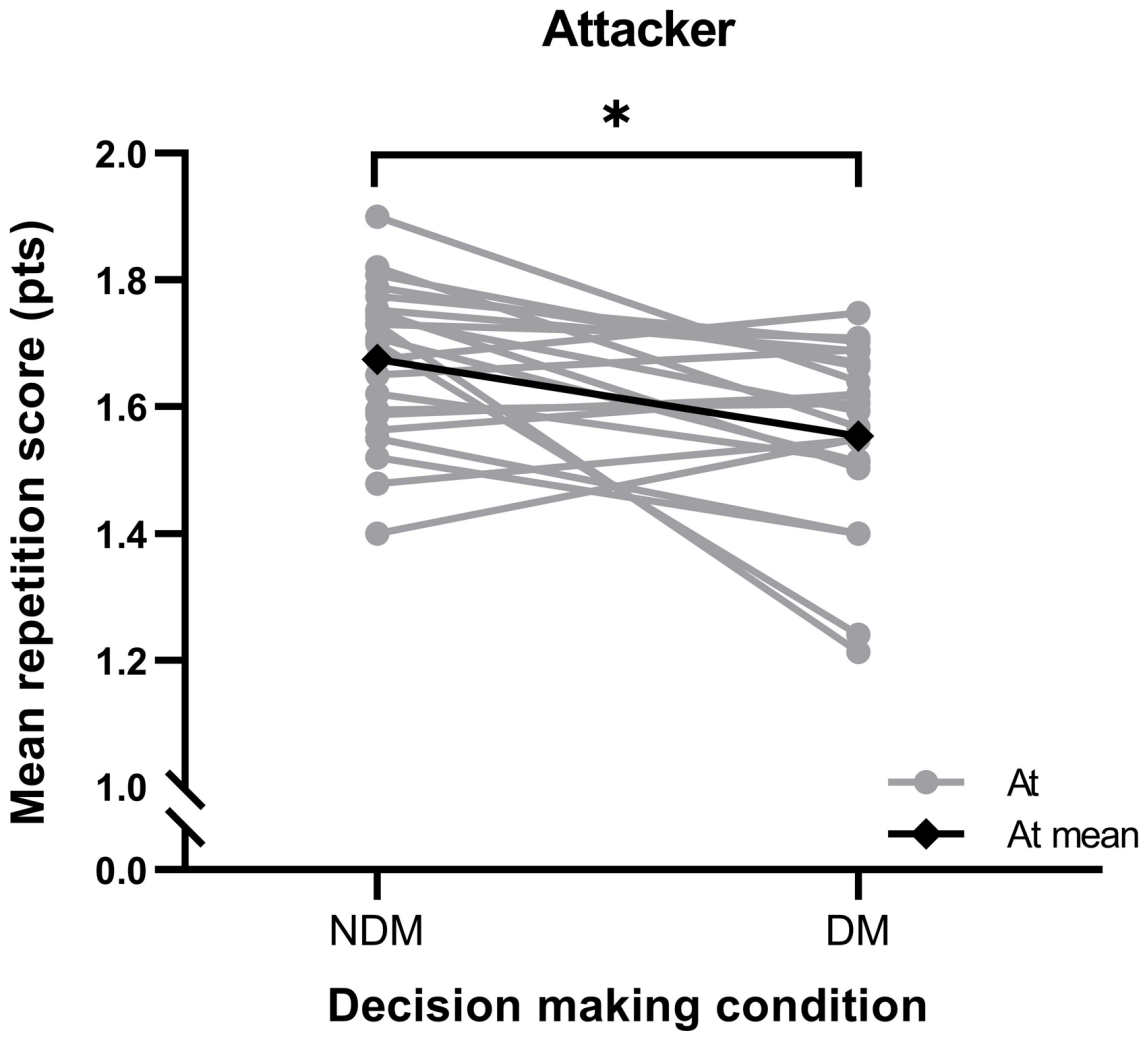
Individual passing accuracy described using mean repetition score of the attacking role and the mean of the role, comparing the no decision making (NDM) and decision making (DM) conditions. The significant differences are shown as * (*p* < 0.01). At: attacker; pts.: points.

Concerning the passing accuracy of the attacker, it was decreased in DM compared to NDM, with a moderate ES (V = 208.5, *p* < 0.01, ES = −0.68, 95%CI: −1.29 – −0.08) (see Figure 5). Moreover, no correlation between MV and passing accuracy of the attacking player was found, in either NDM (*r* = 0.098) or DM (*r* = −0.033). Therefore, it has been shown that decision making did not affect the MV of the attacker but did affect her passing accuracy. Finally, no differences were found when comparing the first team with the second team in MV (*t* = −1.716; *p* = 0.101 in attacker and *t* = −1.176; *p* = 0.253 in defender) and neither in passing accuracy (*t* = −0.358; *p* = 0.724 in attacker).

## 4. Discussion

This study aimed to investigate the effects of decision making of high-level female football players on MV and performance during an elastic band resistance task. The main findings suggested that adding decision making to a forward-backward movement task with ball and elastic resistance increased the MV of the defender but did not affect the MV of the attacker. In contrast, the passing accuracy of the attacker was reduced. Overall, the attacker had a higher MV compared to the defender.

Surprisingly, there were no differences in MV between NDM and DM in the attacker, but there were differences in MV in the defender. For the attacking role, adding a decision making to the task performed (i.e., DM condition) did not significantly increase MV compared to the NDM condition, although a slight upward trend was observed (see Figures 2, 3). Finding no differences could indicate that the incorporation of decision making, which increases the uncertainty of pass direction (Vilar et al., 2012), did not make the task sufficiently complex to achieve a modification of movement regularity. Although the defender performed a defensive action similar to a game situation, the decision making of the attacker can be considered simple, due to reduced contextual information and limited response options (Czyż, 2021). Incorporating decision making into the task presumably involves the exploration and selection of information, which confers characteristics of a real game situation (Williams et al., 1999; Araújo et al., 2007). However, this was not sufficient to alter the regularity of the movement. Even so, it cannot be claimed that both conditions (NDM and DM) had similar signal structure and/or complexity. At the same time, the fact that in the DM condition the players have not experienced an increase in MV could also be due to a possible anticipation of the attacker, taking into account that at the high level, players are able to make accurate predictions from the body movement of their opponents based on postural cues, such as the hip movement (Causer et al., 2017). To avoid anticipation, the opponent should hide his/her postural cues as much as possible. In addition, it is probable that the MV of the attacker could be increased through more complex decision making by adding contextual information (Murphy et al., 2019), for example, by increasing the number of players in the task, or by promoting changes in the interpersonal distance between the ball carrier and the second defender, among others (Travassos et al., 2012a). This would force information filtering in a bottleneck form, as assumed by theories of attention and information processing in parallel order (Broadbent, 1958). In this case, a larger number of action options would be generated than in the DM condition of the present study, which could compromise the MV of the attacker (Murphy et al., 2019). Another explanation for the similar MV results obtained when comparing DM and NDM could be the fact that the body has mechanisms to maintain regularity of movement in the presence of certain constraints (Dhawale et al., 2017). Travassos et al. (2012b), reported changes in regularity of the ball velocity when passes were emergent (i.e., with a large number of possibilities for action), in comparison with predetermined passes. This could indicate that uncertainty on passing direction affects the ball more than the human body.

Regarding the defender, differences in MV were found when decision making was introduced, compared to NDM. In the NDM condition, the players moved forwards and backwards linearly, following the rhythm of the attacker, without the need to adjust their position according to the ball or the opponent. In contrast, in the DM condition, the defender aimed to intercept the ball once the attacker made the pass; therefore, she had to adapt her position and movement to the ball and to her opponent, which was reflected in less regularity of movement. In this condition, the task simulated a 1vs.1 situation of the game, in which the attacker and her marking defender operate as a dyad, considered as a dynamic system with emergent interactions triggered by the interrelation of constraints. Therefore, the movements made by the attacker with the aim of altering the stability of the dyadic system probably affected the defender, increasing the MV (*cf.* Araújo et al., 2006; Davids et al., 2006). Along the same lines, the study developed by Corrêa et al. (2016) suggested that the ball carrier was more successful in dribbling when the interpersonal distance with his marking defender, as well as the angle between the passing and intercepting vector were more variable. The authors noted that this large variability could lead to greater unpredictability for the defending player and, consequently, greater complexity in his/her decision making about how to avoid the dribbling of the attacker.

The attacker showed a higher MV than the defender, with and without decision making. These results can be attributed, in part, to the fact that the attacker, in contrast to the defender, had to pass the ball. The ball pass, which was made with a single touch, involves an interception of a moving object. For a correct interception, it is necessary to perceive and anticipate the trajectory of the ball by capturing information about its position, the direction, and velocity of its displacement, and the acceleration or deceleration it undergoes in its path (Stone et al., 2015). In the present study, the attacker had to perform a continuous forward-backward movement with an elastic resistance in order to make a successful pass. For this purpose, she had to coordinate the starting moment of the approach run, and adjust her velocity and direction to adapt to the trajectory and velocity of the pass made by the staff member. At the same time, she also had to orient her body to immediately make the pass to the chosen or pre-planned side. As the attacker and defender obtained similar RMS ACC and mean repetition time, it can be affirmed that the higher MV obtained by the attacker reflects this perceptual-motor process. The increase in MV found by Moras et al. (2018) when incorporating a rugby-specific ball pass to a movement with inertial resistance was attributed to a change in the coordination patterns of the system or a combination of movement stability and adaptability that induced this specific constraint. Therefore, it could be considered that the interaction with the ball results in a detriment to the movement control or coordination of the athlete (Moras et al., 2018), and this occurs regardless of whether the pass is made with the upper or lower limbs.

Decision making affected the attackers, impairing their passing accuracy. This result could be expected if we consider that the attacking player had to manage more information in the DM condition. In this condition, interrelationships between attacker and defender and the decision making constraint were closer to the competitive performance environment than in the NDM condition, although the possibilities of action and, consequently, the uncertainty of the direction of the pass were limited. The similarity of the DM task to a real game could be also improved through the participation and interaction of a larger number of players. It seems that the addition of a second defender may influence passing accuracy even more than the immediate defender, due to changes in their interpersonal distance (Travassos et al., 2012a). These aspects expand the contextual information, entailing greater cognitive effort and attentional load (Czyż, 2021). This forces players to constantly explore the environment to discover the best action solution based on the immediate demands of the task, compromising their technical accuracy (Travassos et al., 2012a), and slowing down their reaction time (Mudric et al., 2015). The fact that the defender acts directly to steal the ball in the DM condition promotes the adoption of an external focus of attention (Chua et al., 2019), constraining the search space and altering the perception-action coupling employed in the task, which alters the affordances perceived (Pacheco et al., 2019). Natsuhara et al. (2020) reported that in attacking situations with multiple players, 5 vs. 4, and no external resistance, the player receiving and passing the ball mainly paid attention to the attacker-opponent-space relationship. Similarly, the attacker in the task performed in the present study could have had the focus of attention on these external elements. Furthermore, before and during the execution of the task, the attacking players also had to perceive and anticipate the trajectory of the pass made by the staff member, adapt to it, while orienting their body or part of it, in order to make the pass to the chosen side. The attentional diversification could imply a discontinuous information acquisition that must be finally integrated to structure the motor response (Stone et al., 2015). In contrast, in the NDM condition, in the absence of opposition, the attentional strategy could have been different. The attacker probably focused more attention on her own execution and body position than in DM, although she also paid attention to the ball and the target, which may have contributed to the achievement of a higher passing accuracy. Nevertheless, it should be noted that tasks that promote external focus of attention enhance motor learning of athletes (Waite et al., 2022). The lower performance obtained in the DM condition indicates that passing efficacy is reduced when a decision making is added, so it could be considered that the DM condition was more complex for the attacking role than the NDM condition. Reduced passing accuracy should never be considered as a negative factor but should be considered as a necessity to promote the progress of athletes. However, in reinforced-based tasks, an appropriate balance between hits-misses must be established to favor learning (Dhawale et al., 2017).

In the present study, no correlation between MV and passing accuracy of the attacking player was found, in either NDM or DM. Urbán et al. (2019) also found no correlation between variability structure and performance in a cyclic pointing movement task when it was constrained by the target and the reward. Although the relationship between MV and performance depends on the nature of the intrinsic dynamics of the system and the constraints of the task (Newell and Vaillancourt, 2001), the results obtained in the present study could be related to the players’ expertise level. Evidence shows that expert athletes tend to be more regular in their movements than novices (Davids et al., 2008; Bashford et al., 2022), and furthermore, the practice of a movement or motor skill tends to reduce its MV (Fernández-Valdés et al., 2020; Sutter et al., 2022), which can be used as an indicator of sport expertise. In this sense, it could be expected that the group of high-level female football players included in the present study, belonging to the first and second teams, would show differences in MV and passing accuracy when comparing both teams. Although no differences were found in either variable, the t-test and mean comparisons showed a downward trend in the MV of the first team compared to that of the second team, in line with previous studies (Davids et al., 2008; Bashford et al., 2022). In addition, the players in the current study occupied different playing positions, which implies an inconsistent level of specific motor skills. In this sense, in the study of Fernández-Valdés et al. (2022), conducted with rugby players, differences in MV in tackling were found when comparing players from two playing positions, forwards and backwards. Therefore, it is not surprising that the players in the present study achieved different passing accuracy scores with different MV values. However, the potential relationship between years of expertise and/or playing position with the MV obtained in the research task has not been examined. What can be affirmed is that there are different ways of facing the task, with a relationship between MV and passing accuracy specific to each player, which assumes the existence of individualized and identifiable movement patterns (Couceiro et al., 2013; Urbán et al., 2019). In broad terms, four groups can be defined according to the relationship between passing accuracy and MV. (1) A first group with a high level of accuracy and low MV: It corresponds to the players with greater mastery of the task. The low MV would indicate a reduced room for improvement, thus resulting in poor trainability of the task, which does not favor the achievement of new training adaptations. In this case, some authors suggest modifying the task or adding constraints to increase its complexity (Moras et al., 2018). (2) A second group with a high level of accuracy and high MV: In this case, despite successfully achieving the task objective of passing the ball to the receivers with high accuracy, the trainability of the task would be high, as there would be room for improvement in terms of control and regularity of the movement. (3) A third group with a low level of accuracy and high MV: For these players, the task would be excessively challenging, as they not only failed to achieve the task objective successfully but also did not reach a high level of control and regularity of movement. In this case, it would be appropriate to continue training to improve performance and decrease MV, although in certain players it may be necessary to decrease the difficulty or complexity of the task (Fernández-Valdés et al., 2020; Sutter et al., 2022). Finally, (4) a fourth group with a low level of accuracy and low MV: These players would not have mastered the task to achieve their goal successfully and performed it with very regular and rigid movement, which is sometimes associated with a pathological situation or a history of injury (Stergiou and Decker, 2011). Nevertheless, it is worth noting that most of the players in this study belonged to the second group, with high accuracy and high MV. The high MV could be explained by the fact that, although the task was based on known and well-trained skills, the addition of the elastic resistance was a new challenge that altered their motor control. Considering that the effectiveness of resistance training to improve sport performance depends, in part, on motor control and coordination (i.e., MV) and task performance (i.e., passing accuracy), a high MV could be considered beneficial to enhance motor adaptations, because more of the task space is explored (Dhawale et al., 2017).

## 5. Conclusions and practical applications

Introducing decision making to a forward-backward movement task involving ball and elastic resistance resulted in increased MV for the defender and compromised the passing accuracy of the attacker. Overall, the attacker showed a higher MV than the defender. These findings suggest that the inclusion of decision making as a football-specific constraint can enhance the trainability or potential of an elastic resistance task. It is a useful strategy to reduce the control and movement regularity of the defensive role player, while increasing the complexity of the task for the attacking role player. Both effects can prove beneficial in promoting player development.

This type of tasks can be used to implement training with a stronger dynamic correspondence to sport actions, integrating conditional, coordinative, and cognitive abilities, with the aim of improving performance or optimizing the readaptation process, especially in the final stages of injury recovery. Moreover, the MV together with the passing accuracy allow to determine the mastery level of the players, as well as to define the task, which can be used for the individualized prescription of training. Finally, it should be considered beneficial to switch player roles in tasks with external resistance and decision making in order to enhance the players’ adaptive capacity, regardless of their playing position in competition.

## Data availability statement

The datasets generated during the current study are included in the article/Supplementary material, further inquiries can be directed to the corresponding author.

## Ethics statement

The studies involving humans were approved by Ethics Committee for Clinical Research of the Catalan Sports Council. The studies were conducted in accordance with the local legislation and institutional requirements. The participants provided their written informed consent to participate in this study.

## Author contributions

ST and GM conceived and designed the study. ST, GM, JG, and CP-CB conducted the test and collected the data. ST and BF-VV processed the data. ST and GM did the statistical analysis and wrote the original draft. GM supervised all the process. All the authors interpreted the results, revised, edited, and approved the final version of the manuscript.

## Funding

The present study was supported by the Institut Nacional d’Educació Física de Catalunya (INEFC) of the Generalitat de Catalunya and TecnoCampus of the Universitat Pompeu Fabra and by the research groups Grup de Recerca en Activitat Física, Alimentació i Salut (GRAFAiS, Generalitat de Catalunya 2021SGR/01190) and Research group in Technology Applied to High Performance and Health. ST is the recipient of a predoctoral fellowship (PRE107/21/000002).

## Conflict of interest

The authors declare that the research was conducted in the absence of any commercial or financial relationships that could be construed as a potential conflict of interest.

## Publisher’s note

All claims expressed in this article are solely those of the authors and do not necessarily represent those of their affiliated organizations, or those of the publisher, the editors and the reviewers. Any product that may be evaluated in this article, or claim that may be made by its manufacturer, is not guaranteed or endorsed by the publisher.
