# Supplementary material for: Decision making influences movement variability and performance of high-level female football players in an elastic resistance task
Source: Front Psychol. 2023 Sep 15;14:1175248. doi: 10.3389/fpsyg.2023.1175248 (PMC10542582; doi:10.3389/fpsyg.2023.1175248)
Supplement: Supplementary file 1 [file Data_Sheet_1.PDF]

Table S1. Data of all the players with the role of attacker in NDM and DM conditions of the research task. Values of sample entropy, passing accuracy, root mean square acceleration and mean repetition time (mean ± SD) are presented.

		Attacker in NDM				Attacker in DM			
		SampEn (a.u.)	Passing Accuracy (points)	RMS ACC (g)	Mean Repetition Time (s)	SampEn (a.u.)	Passing Accuracy (points)	RMS ACC (g)	Mean Repetition Time (s)
**Players**	**1**	0,170 ± 0,023	1,587 ± 0,248	0,144 ± 0,017	1,925 ± 0,144	0,174 ± 0,003	1,617 ± 0,278	0,152 ± 0,010	1,883 ± 0,059
	**2**	0,168 ± 0,017	1,900 ± 0,141	0,165 ± 0,052	1,850 ± 0,096	0,180 ± 0,010	1,640 ± 0,000	0,159 ± 0,041	2,021 ± 0,032
	**3**	0,170 ± 0,012	1,520 ± 0,243	0,121 ± 0,019	1,777 ± 0,083	0,189 ± 0,038	1,400 ± 0,260	0,260 ± 0,206	1,776 ± 0,105
	**4**	0,161 ± 0,008	1,730 ± 0,090	0,127 ± 0,007	2,086 ± 0,025	0,169 ± 0,016	1,213 ± 0,098	0,130 ± 0,002	2,070 ± 0,337
	**5**	0,140 ± 0,019	1,753 ± 0,113	0,140 ± 0,015	2,032 ± 0,073	0,153 ± 0,016	1,688 ± 0,344	0,134 ± 0,033	1,890 ± 0,138
	**6**	0,170 ± 0,028	1,788 ± 0,132	0,102 ± 0,025	1,888 ± 0,137	0,159 ± 0,020	1,670 ± 0,212	0,110 ± 0,013	2,019 ± 0,186
	**7**	0,202 ± 0,012	1,730 ± 0,156	0,155 ± 0,012	1,975 ± 0,060	0,174 ± 0,008	1,708 ± 0,045	0,133 ± 0,003	2,071 ± 0,301
	**8**	0,192 ± 0,018	1,774 ± 0,120	0,170 ± 0,101	2,048 ± 0,188	0,185 ± 0,005	1,703 ± 0,203	0,107 ± 0,038	1,951 ± 0,251
	**9**	0,165 ± 0,000	1,550 ± 0,000	0,090 ± 0,000	1,924 ± 0,000	0,174 ± 0,000	1,400 ± 0,000	0,102 ± 0,000	2,261 ± 0,000
	**10**	0,142 ± 0,000	1,700 ± 0,000	0,185 ± 0,000	1,835 ± 0,000	0,178 ± 0,023	1,240 ± 0,226	0,155 ± 0,001	1,841 ± 0,044
	**11**	0,131 ± 0,009	1,563 ± 0,100	0,147 ± 0,035	1,815 ± 0,167	0,118 ± 0,010	1,620 ± 0,277	0,118 ± 0,026	1,766 ± 0,032
	**12**	0,160 ± 0,000	1,400 ± 0,000	0,145 ± 0,000	1,890 ± 0,000	0,167 ± 0,000	1,550 ± 0,000	0,181 ± 0,000	1,864 ± 0,000
	**13**	0,178 ± 0,012	1,820 ± 0,127	0,172 ± 0,030	1,833 ± 0,147	0,185 ± 0,010	1,568 ± 0,264	0,139 ± 0,017	1,988 ± 0,051
	**14**	0,166 ± 0,010	1,808 ± 0,055	0,135 ± 0,018	1,837 ± 0,116	0,184 ± 0,009	1,663 ± 0,086	0,159 ± 0,021	1,795 ± 0,180
	**15**	0,175 ± 0,018	1,595 ± 0,090	0,119 ± 0,032	1,806 ± 0,152	0,155 ± 0,007	1,605 ± 0,189	0,136 ± 0,023	1,896 ± 0,096
	**16**	0,177 ± 0,019	1,708 ± 0,177	0,120 ± 0,021	1,753 ± 0,109	0,185 ± 0,009	1,513 ± 0,117	0,239 ± 0,212	1,788 ± 0,109
	**17**	0,201 ± 0,029	1,650 ± 0,264	0,109 ± 0,056	1,757 ± 0,136	0,202 ± 0,006	1,690 ± 0,131	0,117 ± 0,043	1,803 ± 0,168
	**18**	-	-	-	-	0,171 ± 0,015	1,335 ± 0,092	0,129 ± 0,013	1,650 ± 0,109
	**19**	0,185 ± 0,013	1,478 ± 0,320	0,260 ± 0,169	1,859 ± 0,138	0,207 ± 0,023	1,548 ± 0,132	0,123 ± 0,013	1,685 ± 0,117
	**20**	0,162 ± 0,021	1,620 ± 0,302	0,190 ± 0,118	1,777 ± 0,155	0,163 ± 0,013	1,515 ± 0,256	0,242 ± 0,182	1,779 ± 0,088
	**21**	0,184 ± 0,011	1,675 ± 0,171	0,106 ± 0,060	1,961 ± 0,123	0,177 ± 0,006	1,748 ± 0,152	0,070 ± 0,029	1,837 ± 0,159
	**22**	0,149 ± 0,030	1,750 ± 0,229	0,260 ± 0,183	1,971 ± 0,109	0,165 ± 0,013	1,503 ± 0,155	0,156 ± 0,070	2,049 ± 0,282
	**23**	0,191 ± 0,014	1,740 ± 0,295	0,255 ± 0,241	1,859 ± 0,046	0,189 ± 0,014	1,593 ± 0,191	0,145 ± 0,021	1,895 ± 0,085
**Total**		0,170 ± 0,017	1,674 ± 0,178	0,155 ± 0,064	1,884 ± 0,116	0,174 ± 0,013	1,553 ± 0,177	0,148 ± 0,048	1,895 ± 0,140

*NDM: research task without decision making; DM: research task with decision making; SampEn: sample entropy; RMS ACC: root mean square of the total acceleration without gravity.

Table S2. Data of all the players with the role of defender in NDM and DM conditions of the research task. Values of sample entropy, root mean square acceleration and mean repetition time (mean ± SD) are presented.

		Defender in NDM			Defender in DM		
		**SampEn (a.u.)**	**RMS ACC (g)**	**Mean Repetition Time (s)**	**SampEn (a.u.)**	**RMS ACC (g)**	**Mean Repetition Time (s)**
**Players**	**1**	0,119 ± 0,014	0,126 ± 0,007	1,777 ± 0,007	0,161 ± 0,044	0,116 ± 0,009	1,832 ± 0,148
	**2**	0,148 ± 0,005	0,130 ± 0,054	1,879 ± 0,038	0,154 ± 0,025	0,128 ± 0,032	1,940 ± 0,061
	**3**	0,143 ± 0,000	0,130 ± 0,000	1,832 ± 0,000	0,152 ± 0,015	0,139 ± 0,023	1,849 ± 0,065
	**4**	0,108 ± 0,009	0,093 ± 0,016	2,036 ± 0,119	0,152 ± 0,012	0,105 ± 0,025	1,934 ± 0,051
	**5**	0,120 ± 0,018	0,123 ± 0,023	1,868 ± 0,102	0,135 ± 0,041	0,233 ± 0,187	1,998 ± 0,245
	**6**	0,105 ± 0,009	0,069 ± 0,031	2,027 ± 0,104	0,138 ± 0,014	0,075 ± 0,011	1,945 ± 0,111
	**7**	0,119 ± 0,007	0,116 ± 0,046	1,922 ± 0,219	0,148 ± 0,013	0,098 ± 0,038	1,991 ± 0,387
	**8**	0,165 ± 0,014	0,111 ± 0,024	2,039 ± 0,074	0,168 ± 0,020	0,094 ± 0,027	2,332 ± 0,145
	**9**	-	-	-	0,176 ± 0,002	0,117 ± 0,047	1,784 ± 0,188
	**10**	0,129 ± 0,000	0,069 ± 0,000	2,001 ± 0,000	0,135 ± 0,005	0,185 ± 0,158	1,875 ± 0,194
	**11**	0,128 ± 0,008	0,126 ± 0,017	1,991 ± 0,142	0,173 ± 0,017	0,125 ± 0,036	1,862 ± 0,058
	**12**	0,106 ± 0,000	0,176 ± 0,000	1,677 ± 0,000	0,152 ± 0,000	0,138 ± 0,000	1,784 ± 0,000
	**13**	0,153 ± 0,016	0,096 ± 0,027	1,837 ± 0,196	0,181 ± 0,024	0,194 ± 0,206	1,904 ± 0,108
	**14**	0,133 ± 0,009	0,158 ± 0,062	1,857 ± 0,151	0,169 ± 0,015	0,138 ± 0,009	1,873 ± 0,154
	**15**	0,131 ± 0,006	0,177 ± 0,119	1,789 ± 0,135	0,145 ± 0,012	0,126 ± 0,043	1,824 ± 0,058
	**16**	0,143 ± 0,018	0,104 ± 0,018	1,803 ± 0,113	0,153 ± 0,026	0,145 ± 0,086	1,767 ± 0,266
	**17**	0,169 ± 0,004	0,180 ± 0,072	1,762 ± 0,211	0,201 ± 0,015	0,190 ± 0,119	1,851 ± 0,119
	**18**	0,150 ± 0,000	0,306 ± 0,000	1,718 ± 0,000	0,136 ± 0,000	0,137 ± 0,000	1,530 ± 0,000
	**19**	0,144 ± 0,018	0,132 ± 0,091	1,780 ± 0,095	0,184 ± 0,015	0,091 ± 0,021	1,729 ± 0,093
	**20**	0,107 ± 0,006	0,196 ± 0,131	1,885 ± 0,095	0,131 ± 0,011	0,212 ± 0,148	1,785 ± 0,161
	**21**	0,140 ± 0,006	0,121 ± 0,004	1,989 ± 0,127	0,144 ± 0,022	0,097 ± 0,013	1,935 ± 0,318
	**22**	0,130 ± 0,005	0,139 ± 0,036	1,974 ± 0,103	0,150 ± 0,022	0,102 ± 0,012	1,860 ± 0,160
	**23**	0,148 ± 0,012	0,265 ± 0,216	1,856 ± 0,087	0,169 ± 0,020	0,113 ± 0,013	1,881 ± 0,040
**Total**		0,134 ± 0,010	0,143 ± 0,055	1,877 ± 0,118	0,157 ± 0,019	0,135 ± 0,060	1,872 ± 0,149

*NDM: research task without decision making; DM: research task with decision making; SampEn: sample entropy; RMS ACC: root mean square of the total acceleration without gravity.
